# Toward Long-Term-Dispersible, Metal-Free Single-Chain Nanoparticles

**DOI:** 10.3390/nano13081394

**Published:** 2023-04-18

**Authors:** Agustín Blázquez-Martín, Ainara Ruiz-Bardillo, Ester Verde-Sesto, Amaia Iturrospe, Arantxa Arbe, José A. Pomposo

**Affiliations:** 1 Centro de Física de Materiales (CSIC, UPV/EHU)-Materials Physics Center (MPC), 20018 Donostia-San Sebastián, Spainarantxa.arbe@ehu.eus (A.A.); 2 IKERBASQUE-Basque Foundation for Science, 48009 Bilbao, Spain; 3 Department of Polymers and Advanced Materials: Physics, Chemistry and Technology, University of the Basque Country UPV/EHU, 20018 Donostia-San Sebastián, Spain

**Keywords:** single-chain nanoparticles (SCNPs), metal-free click chemistry, intramolecular cross-linking, *sym*-dibenzo-1,5-cyclooctadiene-3,7-diyne (DIBOD), strain-promoted azide−alkyne cycloaddition (SPAAC)

## Abstract

We report herein on a new platform for synthesizing stable, inert, and dispersible metal-free single-chain nanoparticles (SCNPs) via intramolecular metal-traceless azide–alkyne click chemistry. It is well known that SCNPs synthesized via Cu(I)-catalyzed azide–alkyne cycloaddition (CuAAC) often experience metal-induced aggregation issues during storage. Moreover, the presence of metal traces limits its use in a number of potential applications. To address these problems, we selected a bifunctional cross-linker molecule, *sym*-dibenzo-1,5-cyclooctadiene-3,7-diyne (DIBOD). DIBOD has two highly strained alkyne bonds that allow for the synthesis of metal-free SCNPs. We demonstrate the utility of this new approach by synthesizing metal-free polystyrene (PS)-SCNPs without significant aggregation issues during storage, as demonstrated by small-angle X-ray scattering (SAXS) experiments. Notably, this method paves the way for the synthesis of long-term-dispersible, metal-free SCNPs from potentially any polymer precursor decorated with azide functional groups.

## 1. Introduction

Single-chain nanoparticles (SCNPs), formed by the folding/collapse of discrete individual polymer chains via intra-chain interactions, are ultra-small soft particles (3–30 nm) that have a variety of applications (catalysis, sensing, drug delivery, nanocomposites) [1,2,3,4,5,6,7,8,9]. Concerning the nature of the intra-chain interactions, permanent SCNPs result when covalent bonds are involved in the intramolecular collapse of the precursor chain, whereas reversible SCNPs are obtained by intra-chain folding through non-covalent interactions or dynamic covalent bonds [10,11,12,13,14,15]. Although reversible SCNPs respond to external stimuli (e.g., temperature, solvents, pH) and find applications in enzyme mimicry and drug delivery [16,17,18,19,20], permanent SCNPs are more appropriate for applications in which long-term stability is a prerequisite such as catalysis or all-polymer nanocomposites (APNCs) [21,22,23,24,25].

Among the different techniques developed to synthesize permanent SCNPs endowed with robust intra-chain covalent bonds, those based on “click” chemistry are regarded as attractive [26]. Since its discovery by Sharpless [27] and Meldal [28] in 2002, Cu(I)-catalyzed azide–alkyne coupling (CuAAC)—the archetypical “click” reaction [29]—has gained enormous interest in a broad number of fields, including pharmaceutical science and materials science. One of the authors of this paper pioneered the use of CuAAC for SCNP construction a few years after its discovery [30,31,32]. CuAAC is a rapid, high-yielding reaction that can be conducted at room temperature (r.t.) using diluted concentrations of reactants, making it compatible with many other functional groups. However, the resulting triazole group formed on CuAAC is prone to complex Cu ions from the catalyst. In this sense, the removal of metal traces from polymers and SCNPs is significantly more complicated than in the case of water-soluble low-molecular-weight substances and, consequently, severely limits practical applications due to potential toxicity, coloration, and stability issues in the resulting materials [33,34]. On the one hand, the interference of Cu ions with many biological processes (for instance, metal ions can strongly bind to guanosine bases, disrupting the double-helical structure of DNA) [35] and various electrical/optoelectronic phenomena [36] is well-known. On the other hand, metal ions in some macromolecule–metal complexes tend to aggregate, forming nanoscale ionic domains due to their electrostatic or dipole–dipole interactions [37]. In the case of SCNPs, these effects lead to the irreversible aggregation of SCNPs into multi-chain nanoparticles during storage in the solid state [38]. Consequently, when SCNPs containing limited amounts of Cu ions are dissolved after storage, aggregates with significantly larger sizes than individual SCNPs are observed.

**Figure 1:**
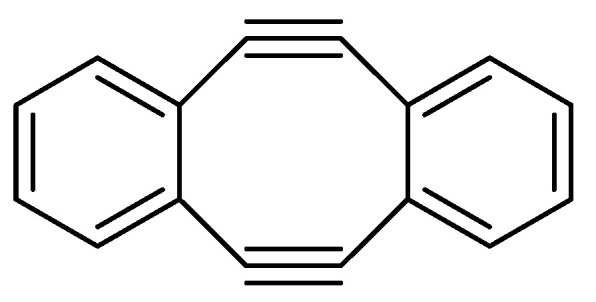
Structure of *sym*-dibenzo-1,5-cyclooctadiene-3,7-diyne (DIBOD).

A promising approach to avoid any residual metal ions while retaining the advantages of click chemistry is to use metal-free versions, such as the strain-promoted azide−alkyne cycloaddition (SPAAC) developed by Bertozzi [39] for covalent modification of biomolecules in living systems. SPAAC utilizes strained cyclooctynes that have a decreased activation energy to react with azides in a click-like fashion, eliminating the need for a Cu(I) catalyst. This type of metal-free click chemistry has been applied in several fields, from dynamic in vivo imaging [40] to cellulose modification [41] and the functionalization of surfaces [42], among others. In this work, in order to synthesize metal-free SCNPs that are free from aggregation issues during storage in the solid state, we turn our attention to a bifunctional cross-linker molecule, *sym*-dibenzo-1,5-cyclooctadiene-3,7-diyne (DIBOD), with two highly strained alkyne bonds(Figure 1). DIBOD was first synthesized by Sondheimer et al. in 1974 [43]. The use of DIBOD for fluorescence labeling of azido-glycoconjugates on cell surfaces was pioneered by Hosoya et al. in 2010 [44]. More recently, this bifunctional cross-linker molecule has been employed for the efficient construction of various rings [45] and cage-shaped polymers [46]. However, to the best of our knowledge, DIBOD has not yet been investigated as an intra-chain cross-linker for metal-free SCNP construction.

To illustrate the utility of DIBOD as an external bifunctional cross-linker molecule for the synthesis of metal-free SCNPs via SPAAC, we first synthetized a polystyrene (PS)-based precursor decorated with azide functional groups. Next, we investigated the reaction conditions to produce PS-SCNPs using DIBOD molecules as intra-chain cross-linkers. Through small-angle X-ray scattering (SAXS) experiments, we found that this new approach leads to metal-free PS-SCNPs without significant aggregation issues during storage. Remarkably, the new synthetic method reported in this work is useful for the preparation of long-term-dispersible, metal-free SCNPs from potentially any polymer precursor decorated with azide functional groups.

## 2. Results and Discussion

**Scheme 1:**
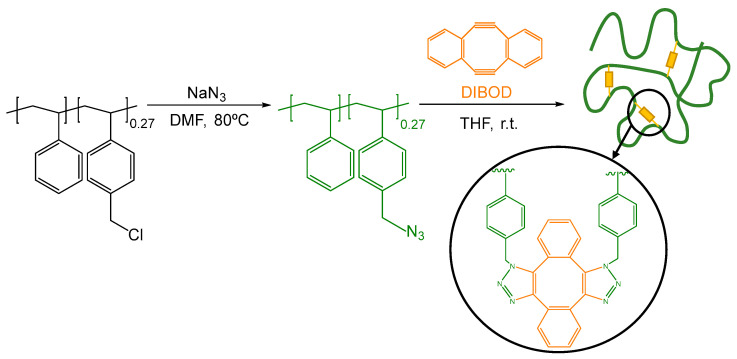
Illustration of the synthesis of metal-free PS-SCNPs, **3**, at r.t. from a PS-based precursor decorated with azide functional groups (**2**).

Scheme 1 illustrates the procedure followed in this work for the synthesis of metal-free PS-SCNPs via SPAAC using DIBOD molecules as intra-chain cross-linkers.

### 2.1. Synthesis and Characterization of the Polystyrene-Based Precursor Decorated with Azide Functional Groups (***2***)

**Figure 2:**
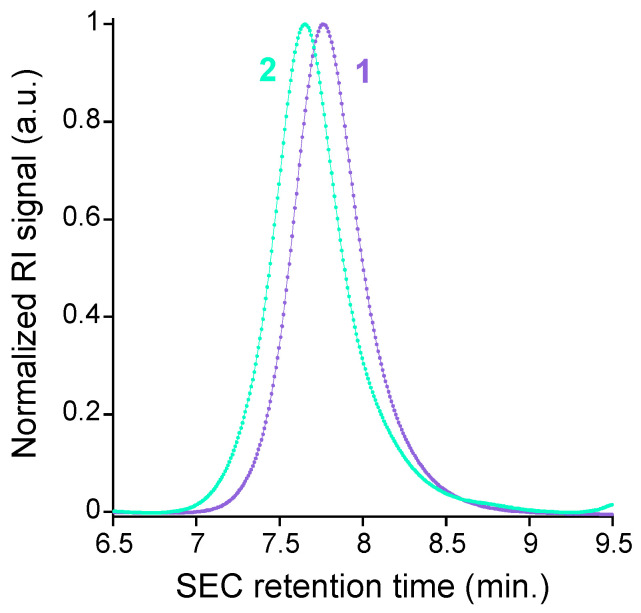
SEC traces (RI detector) of poly(S-co-CMS), **1**, and poly(S-co-AMS), **2**.

In the first step, we synthetized a random copolymer of styrene (S) and chloromethyl styrene (CMS) via reversible addition-fragmentation chain-transfer (RAFT) polymerization (see Scheme 1) [47]. The resulting poly(S-co-CMS) copolymer, **1**, containing 27 mol% of CMS units—as estimated by proton nuclear magnetic resonance (^1^H NMR)—showed a weight-average molecular weight (*M*_w_) of 100.5 kDa and a narrow dispersity (*Ɖ*) of 1.15, as determined by size exclusion chromatography (SEC) equipped with triple detection (RI, MALS, and VIS, see Section 3.2.1). The SEC/RI trace of **1** is illustrated in Figure 2. The hydrodynamic radius (*R*_h_) of **1** in THF was *R*_h_ (VIS) = 8.2 nm.

**Figure 3:**
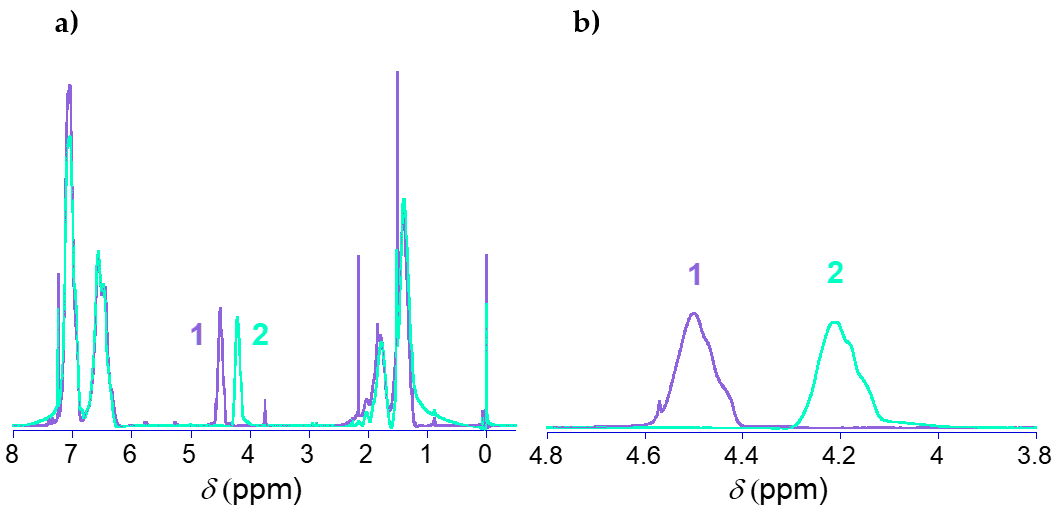
(**a**) ^1^H NMR spectra of poly(S-co-CMS), **1**, and poly(S-co-AMS), **2**. (**b**) Enlarged region between 3.8 and 4.8 ppm showing the shift of the peak corresponding to methylene protons next to –Cl moieties in **1** during transformation to methylene protons next to –N_3_ moieties in **2**. The complete shift observed confirms the successful total replacement of –Cl moieties in **1** with –N_3_ moieties in **2**.

**Figure 4:**
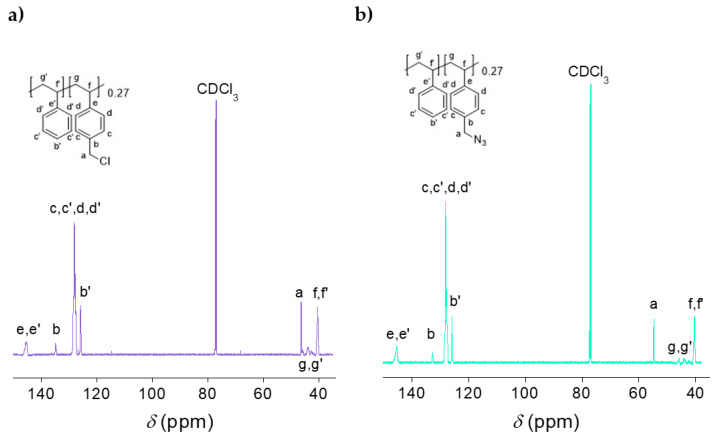
(**a**) ^13^C NMR spectrum of poly(S-co-CMS), **1**. (**b**) ^13^C NMR spectrum of poly(S-co-AMS), **2**. Note the clear shift in the position of the peak corresponding to the methylene carbon (denoted as **a**) during the replacement of all –Cl groups with –N_3_ moieties.

Subsequently, **1** was subjected to an azidation procedure [47] (NaN_3_, DFMF, 24 h) at 80 °C to give the polystyrene (PS)-based precursor decorated with –N_3_ (instead –Cl) functional groups: poly(S-co-AMS), **2** (see Scheme 1). The SEC/RI trace of **2** is shown in Figure 2, with values of *M*_w_ (MALS) = 129.1 kDa, *Ð* = 1.18, and *R*_h_ (VIS) = 9.2 nm. We attributed the slight increase in *R*_h_ to steric effects along the copolymer chain induced by the replacement of the –Cl groups with –N_3_ moieties. The total replacement of –Cl moieties with –N_3_ functional groups was confirmed by both ^1^H and ^13^C NMR spectroscopy, as shown in Figure 3 and Figure 4.

### 2.2. Synthesis and Characterization of Metal-Free Single-Chain Nanoparticles (***3***) from the Polystyrene-Based Precursor Decorated with Azide Functional Groups (***2***)

**Figure 5:**
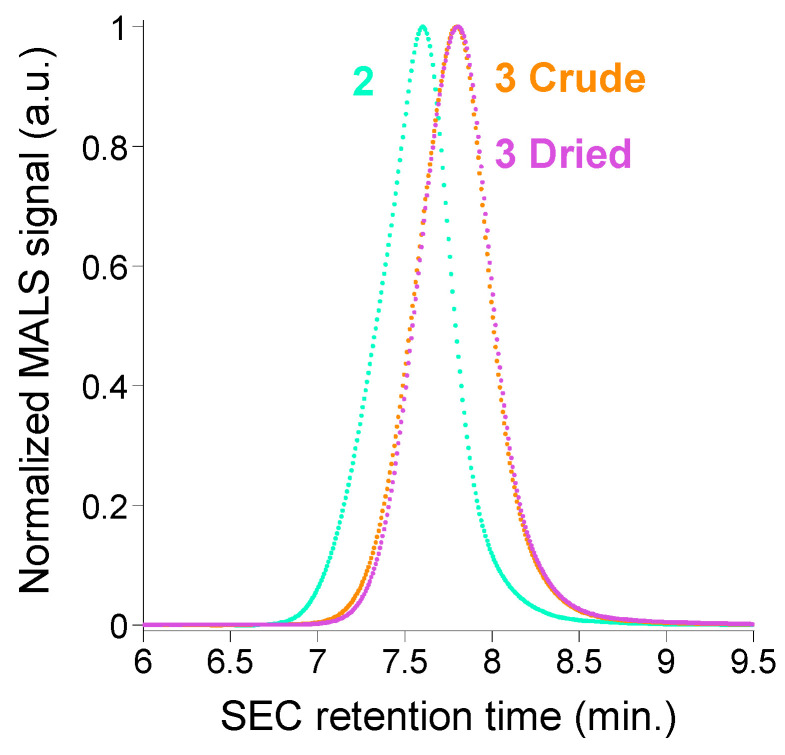
SEC traces (MALS detector) of poly(S-co-AMS), **2**; metal-free PS-SCNPs as synthetized without isolation, **3 Crude**; and metal-free PS-SCNPs after isolation, **3 Dried**.

**Figure 6:**
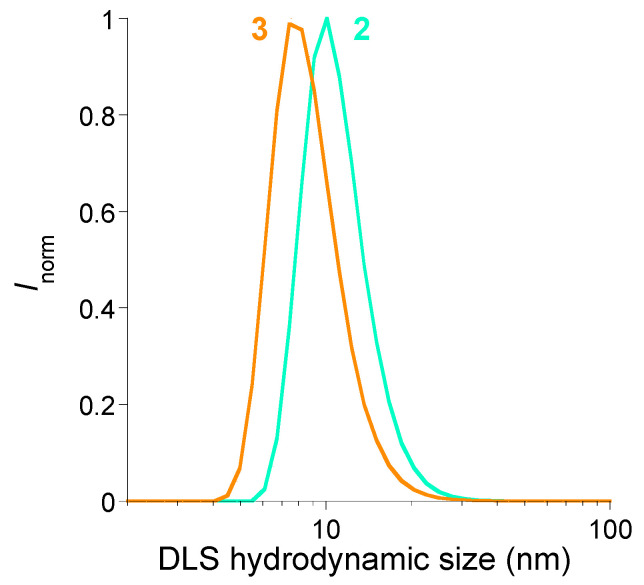
Hydrodynamic size distribution of poly(S-co-AMS), **2**, and metal-free PS-SCNPs, **3**, as determined by dynamic light scattering (DLS) measurements. Precursor **2**: *R*_h_ (DLS) = 10.1 nm. PS-SCNPs, **3**: *R*_h_ (DLS) = 7.5 nm.

Metal-free PS-SCNPs were synthesized at r.t. via SPAAC using DIBOD molecules as intra-chain cross-linkers, as depicted in Scheme 1. We used a slow addition technique, first introduced by Hawker et al. [48], in which a solution of the precursor (i.e., **2**) was added at a low addition rate to a solution of the cross-linker (i.e., DIBOD). After the complete addition of **2** in ca. 12 h, the mixture remained (additionally) for 12 h under stirring to allow the SPAAC to go to completion. Then, an appropriate amount of BzA was added to end-cap any potential unreacted alkyne group, and the mixture was left under stirring for an additional 24 h. After concentrating the reaction mixture, **3** was obtained by precipitation in cold EtOH. Figure 5 shows the SEC/MALS traces of **2**, **3** before precipitation after 48 h of reaction, and **3** after precipitation, drying, and re-dissolution. No change in the SEC trace of the PS-SCNPs, **3**, was observed from the crude to the dried (and re-dissolved) samples. The PS-SCNPs, **3,** exhibited values of *M*_w_ (MALS) = 195.8 kDa, *Ð* = 1.21, and *R*_h_ (VIS) = 6.9 nm. The increase in *M*_w_ observed was due to the incorporation of the intra-chain cross-linker DIBOD. The SEC results confirmed the successful formation of SCNPs with a hydrodynamic size (*R*_h_ (VIS) = 6.9 nm) smaller than that of the precursor copolymer (*R*_h_ (VIS) = 9.2 nm). We can exclude the presence of multi-chain aggregates since the SEC/MALS technique is highly sensitive to their presence, and they should be observed at a low SEC retention time (<7 min.). Additionally, dynamic light scattering (DLS) measurements also showed a significant reduction in size (*R*_h_ (DLS) = 7.5 nm) upon the formation of metal-free PS-SCNPs via SPAAC using DIBOD molecules as intra-chain cross-linkers, as shown in Figure 6.

**Figure 7:**
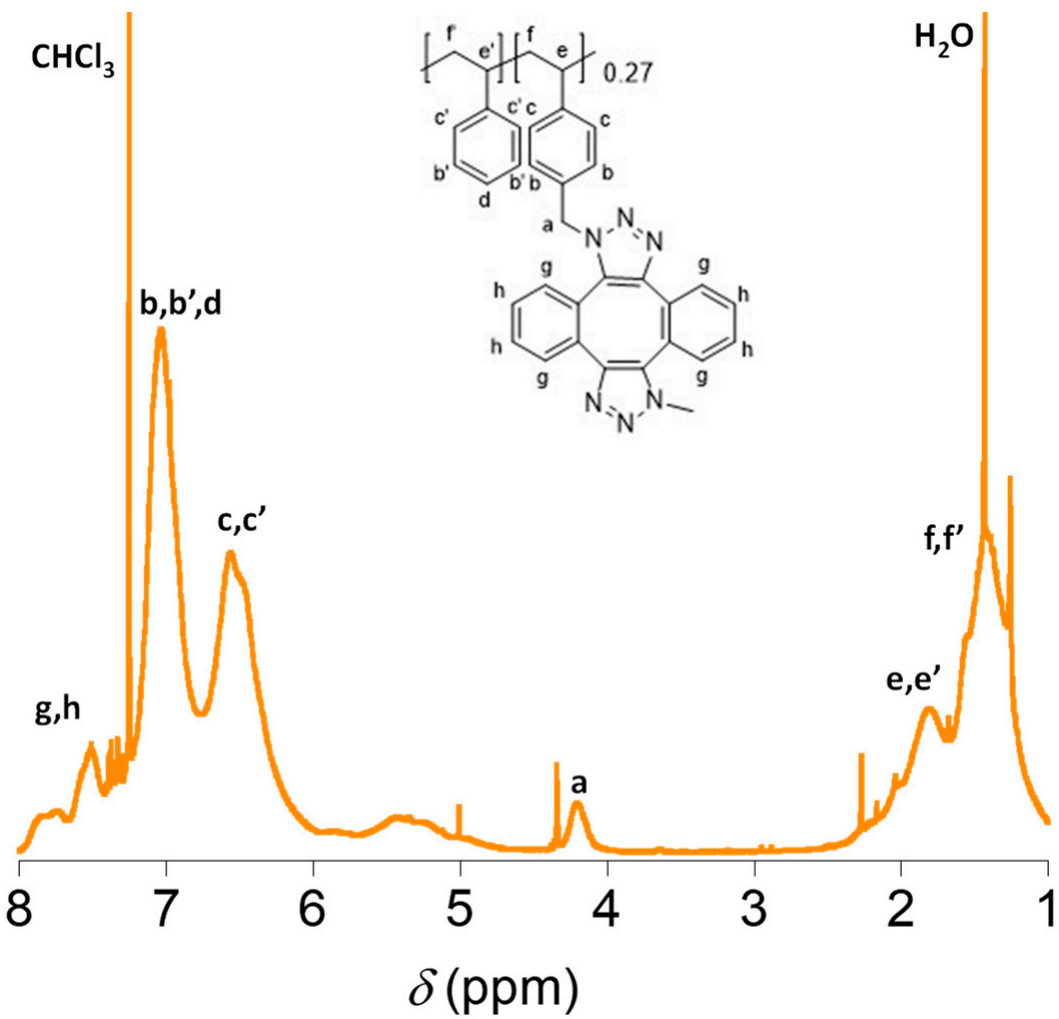
^1^H NMR spectra of metal-free PS-SCNPs, **3**. Peaks denoted as **g** and **h** correspond to aromatic protons from the DIBOD intra-chain cross-linker.

**Figure 8:**
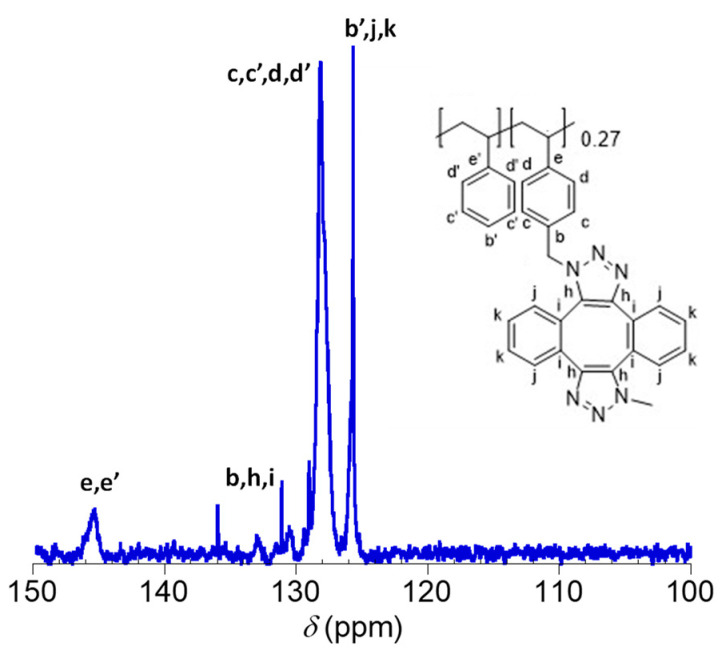
^13^C NMR spectra of metal-free PS-SCNPs, **3**, showing the positions of the different aromatic carbons, including those from the DIBOD intra-chain cross-linker.

**Figure 9:**
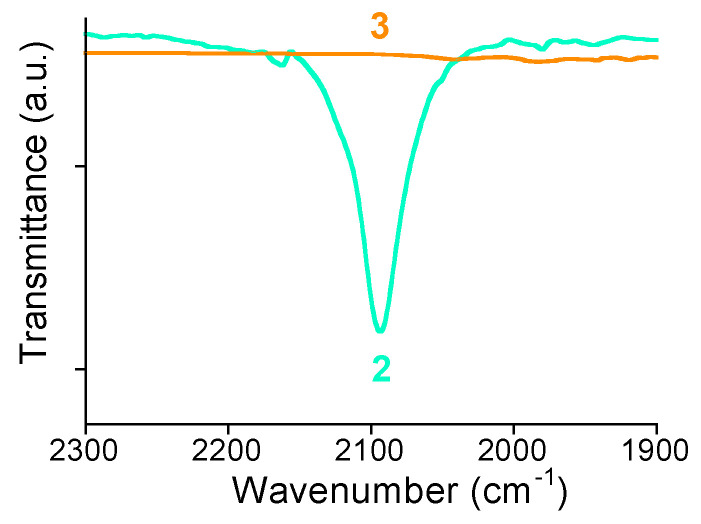
Disappearance of the azide IR vibration band of precursor **2** upon reaction with DIBOD and the formation of metal-free SCNPs, **3**.

The incorporation of DIBOD molecules as intra-chain cross-linkers was confirmed by ^1^H NMR and ^13^C NMR spectroscopy, as illustrated in Figure 7 and Figure 8, respectively. Additionally, the disappearance of the infra-red (IR) vibration band corresponding to the azide functional group located near 2100 cm^−1^ was clearly visible in the FTIR spectrum of the metal-free PS-SCNPs, **3**, as illustrated in Figure 9.

**Figure 10:**
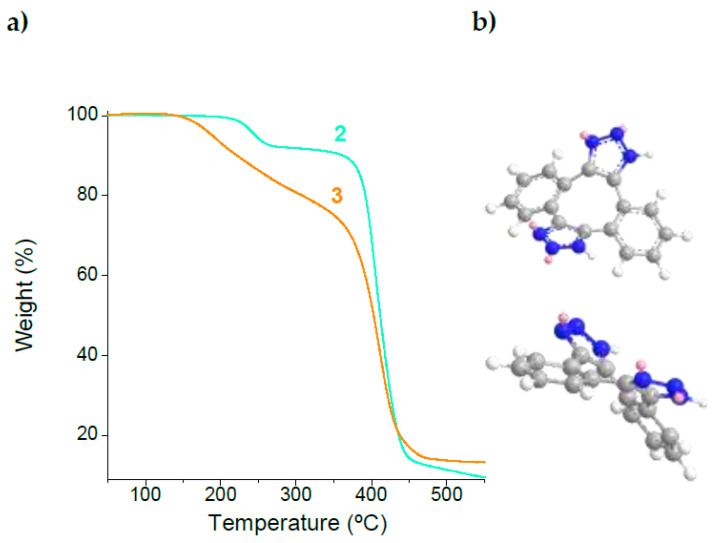
(**a**) Comparison of the thermal stability of precursor **2** and the resulting metal-free SCNPs, **3**. (**b**) The lower thermal stability of **3** can be attributed to the strained nature of the 1,10-dihydrodibenzo [3,4:7,8]cycloocta [1,2-d:5,6-d’]bis([1,2,3]triazole) units of the PS-SCNPs, as revealed by the conformation found after energy minimization (MMFF94 force field, Chem3D).

Figure 10a illustrates the thermal stability of precursor **2** and the PS-SCNPs, **3,** as determined by TGA measurements. The initial decomposition temperature of **2** was 220 °C, corresponding to the extrusion of dinitrogen molecules from the azide groups. The initial decomposition temperature of **3** was found to be 153 °C. We attributed the lower thermal stability of **3** to the strained nature of the 1,10-dihydrodibenzo [3,4:7,8]cycloocta [1,2-d:5,6-d’]bis([1,2,3]triazole) cross-linking units (see Figure 10b), which promoted their thermal decomposition when heating above 150 °C. Below this limiting temperature, the PS-SCNPs showed no weight loss so one can consider **3** to be a stable material up to 150 °C.

### 2.3. Long-Term Stability against Aggregation of Metal-Free Polystyrene-Single-Chain Nanoparticles (***3***)

**Figure 11:**
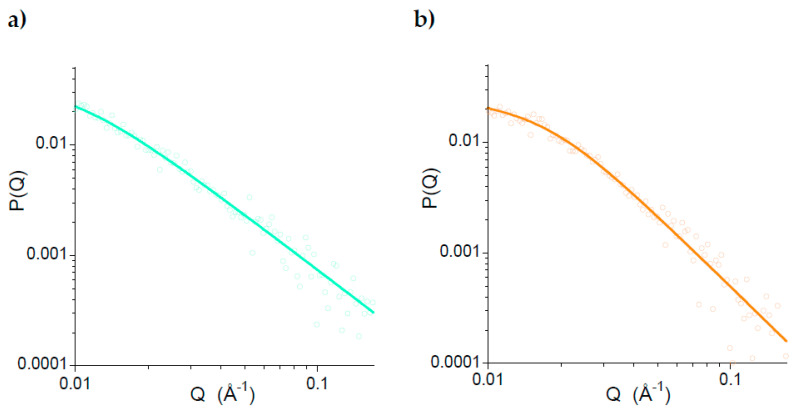
SAXS results revealing: (**a**) the form factor of precursor **2**, and (**b**) the form factor of the metal-free SCNPs, **3**, as synthetized. Values of *R*_g_ and *ν* were obtained through fits of the experimental P(Q) vs. Q data to a generalized Gaussian coil function. Precursor **2**: *R*_g_ = 14.1 nm, *ν* = 0.59. PS-SCNPs, **3**: *R*_g_ = 9.1 nm, *ν* = 0.46.

The long-term stability against aggregation of the metal-free PS-SCNPs was investigated using small angle X-ray scattering (SAXS) measurements in THF solution at high dilution. Reference SAXS measurements were taken to determine the form factor of precursor **2** and the metal-free PS-SCNPs, **3,** after synthesis (see Figure 11). Analysis of the data shown in Figure 11 in terms of the generalized Gaussian coil function [49] provided the values of the radius of gyration (*R*_g_) and scaling exponent (*ν*) of **2** and **3** in solution at high dilution. Precursor **2** showed a size of *R*_g_ = 14.1 nm and *ν* = 0.59, a scaling exponent value that was in agreement with the expected Flory value for a linear chain in good solvent (*ν*_F_ ≈ 3/5). On the other hand, the freshly synthesized metal-free PS-SCNPs exhibited a reduction in both the size and scaling exponent: *R*_g_ = 9.1 nm and *ν* = 0.46. These values were in agreement with previously reported data on the single-chain folding of synthetic polymers to SCNPs [1,38].

**Figure 12:**
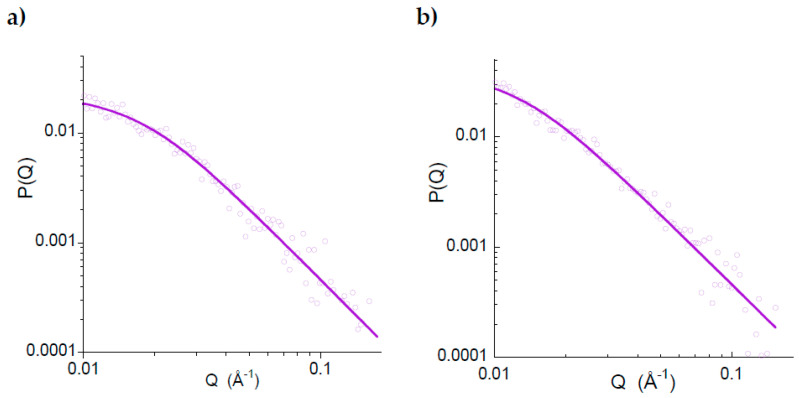
SAXS form factor of: (**a**) **3** after 2 months of storage in THF solution (*R*_g_ = 8.7 nm, *ν* = 0.46), and (**b**) **3** after 2 months of storage in the solid state (*R*_g_ = 12.0 nm, *ν* = 0.47).

Figure 12 shows the SAXS results obtained for the SCNPs, **3,** in THF dilute solution after 2 months of storage. Figure 12a corresponds to storage in the same solution, whereas Figure 12b corresponds to storage in the solid state and the redispersion of the SCNPs in THF. Analysis of the data in terms of a generalized Gaussian coil function revealed no significant changes (within the experimental uncertainties) in *R*_g_ or*ν* with respect to the values observed for the PS-SCNPs, **3**, as synthetized (Figure 11b). Only in the case of storage in the solid state was a minor increase in *R*_g_ observed, although the scaling exponent did not change significantly. Hence, unlike the case of CuAAC in which large aggregates are typically found after prolonged storage, the synthesis of the metal-free SCNPs via SPAAC using DIBOD molecules as intra-chain cross-linkers can prevent irreversible multi-chain aggregation issues.

We are currently investigating other azide-containing precursors besides PS to evaluate the scope of the SPAAC method using DIBOD molecules as intra-chain cross-linkers for the synthesis of long-term-dispersible, metal-free SCNPs. The results will be reported at another time.

## 3. Materials and Methods

### 3.1. Materials

Styrene (S, ≥99%), sodium azide (NaN_3_, ≥99%), N,N-dimethylformamide (DMF, ≥99.9%), and 1,1′-azobis(cyclohexanecarbonitrile) (ACHN, ≥98%) were purchased from Sigma-Aldrich (Madrid, Spain). Chloromethyl styrene (CMS, ≥90%) and *sym*-dibenzo-1,5-cyclooctadiene-3,7-diyne (DIBOD, > 98%) were supplied by TCI. Tetrahydrofuran (THF, GPC grade) and methanol (MeOH, analytical grade) were purchased from Scharlau (Barcelona, Spain). Benzyl azide (BzA, 94%), S-cyanomethyl-S-dodecyltrithiocarbonate (CDTC, ≥97%), and ethanol (EtOH, 96% v/v) were supplied by Thermo Scientific (Eindhoven, Netherlands), Strem (Bischheim, France), and PanReac AppliChem (Barcelona, Spain), respectively. Unless otherwise specified, all reagents were used as received without further purification. To remove the inhibitor, S and CMS were purified by being passed through a column packed with activated basic alumina.

### 3.2. Techniques

#### 3.2.1. Size-Exclusion Chromatography (SEC)

SEC measurements were performed at 30 °C on an Agilent 1200 system equipped with PLgel 5 μm Guard and PLgel 5 μm MIXED-C columns, and triple detection: a differential refractive index (RI) detector (Optilab Rex, Wyatt, Toulouse, France), a multi-angle laser light scattering (MALS) detector (MiniDawn Treos, Wyatt, Toulouse, France), and a viscosimetric (VIS) detector (ViscoStar-II, Wyatt, Toulouse, France). Data analysis was performed using ASTRA Software (version 6.1) from Wyatt. THF was used as an eluent at a flow rate of 1 mL/min. A value of dn/dc = 0.186 was used for poly(S-co-CMS), **1**, poly(S-co-AMS), **2**, and the metal-free PS-SCNPs, **3**.

#### 3.2.2. Nuclear Magnetic Resonance (NMR)

^1^H NMR and ^13^C NMR spectra were recorded at room temperature on Bruker (Madrid, Spain) spectrometers operating at 400 MHz, using CDCl_3_ as solvent.

#### 3.2.3. Dynamic Light Scattering (DLS)

DLS measurements (number distribution) were taken at room temperature on a Malvern Zetasizer Nano ZS (Cambridge, United Kingdom) apparatus.

#### 3.2.4. Fourier Transform Infra-Red (FTIR) Spectroscopy

FTIR spectra were recorded at room temperature on a JASCO 3600 (Madrid, Spain) FTIR spectrometer.

#### 3.2.5. Thermogravimetric Analysis (TGA)

TGA measurements were performed on a Q500-TA Instruments (Cerdanyola del Valles, Spain) apparatus at a heating rate of 10 °C/min under a nitrogen atmosphere.

#### 3.2.6. Small-Angle X-ray Scattering (SAXS)

SAXS experiments were conducted on the Rigaku (Barcelona, Spain) 3-pinhole PSAXS-L equipment of the Materials Physics Center, operating at 45 kV and 0.88 mA. The MicroMax-002+ X-ray Generator System is composed of a microfocus sealed tube source module and an integrated X-ray generator unit, which produces CuKα transition photons of wavelength λ = 1.54 Å. The flight path and the sample chamber in this equipment are under vacuum. The scattered X-rays are detected on a two-dimensional multiwire X-ray Detector (Gabriel (Barcelona, Spain) design, 2D-200X) and converted to one-dimensional scattering curves by radial averaging. This gas-filled proportional type detector offers a 200 mm diameter active area with ca. 200-micron resolution. After radial integration, the scattered intensities were obtained as a function of momentum transfer Q = 4πλ^−1^ sinθ, where θ is half the scattering angle. Reciprocal space calibration was performed using silver behenate as standard. The sample-to-detector distance was 2 m, covering a Q-range between 0.008 and 0.20 Å^−1^. The measurements were taken at r.t. on the THF solutions in boron-rich capillaries of 2 mm thickness, with counting times of 1 h. The concentration was 1 mg/mL in order to avoid interference effects between different macromolecules. The data were carefully corrected for background scattering (due to the capillary and solvent) and measured for each sample on the same capillary for the same time. Scattering cross-sections were obtained in absolute units using water as the calibration standard. The generalized Gaussian coil function [49] was employed for a precise determination of the values of the radius of gyration, *R*_g_, and scaling exponent, *ν*.

### 3.3. Procedures

#### 3.3.1. Synthesis of Poly(S-co-CMS), **1**

P(S-co-CMS), **1**, was prepared by RAFT polymerization in bulk. S (2 mL, 17.4 mmol, 1 eq), CMS (1.05 mL, 7.45 mmol, 0.43 eq), ACHN as the initiators (2.6 mg, 6.12 × 10^−4^ eq), and CDTC (5.1 mg, 9.23 × 10^−4^ eq) as the RAFT agent were added to a 50 mL round-bottom flask. The reaction was stirred at 65 °C for 48 h. P(S-co-CMS), **1,** was obtained by precipitation in MeOH, isolated by filtration, and finally dried in a vacuum oven at 35 °C overnight. Its parameter values were: yield = 40%; CMS content (^1^H NMR) = 27 mol%; *M*_w_ (SEC/MALS) = 100.5 kDa; *Ð* (SEC) = 1.15; and *R*_h_ (SEC/VIS) = 8.2 nm.

#### 3.3.2. Synthesis of Poly(S-co-AMS), **2**

P(S-co-CMS), **2**, (2 g, 4.65 mmol of Cl, 1 eq) and NaN_3_ (0.604 g, 9.3 mmol, 2 eq) were dissolved in DMF (80 mL) in a 100 mL round-bottom flask and the resulting mixture was stirred at 80 °C for 24 h. P(S-co-CMS), **2** was recovered by precipitation in a mixture of MeOH and water (1:1) and then filtered and dried at 35 °C under vacuum overnight. ^1^H and ^13^C NMR spectroscopy were used to check that all the chlorine atoms in **1** were substituted with azide groups. Its parameter values were yield = 97%; azide content (^1^H NMR) = 27 mol%; *M*_w_ (SEC/MALS) = 129.1 kDa; *Ð* (SEC) = 1.18; and *R*_h_ (SEC/VIS) = 9.2 nm.

#### 3.3.3. Synthesis of Metal-Free PS-SCNPs, **3**

Poly(S-co-AMS), **2**, (50 mg, 0.106 mmol of N_3_, 1 eq) was dissolved in THF (25 mL) in a flask that was sealed and degassed. In a second flask that was also sealed, degassed, and protected from light with aluminum foil, DIBOD (12 mg, 0.53 eq) was dissolved in 75 mL of THF. The solution containing **2** was injected into the DIBOD solution (both at r.t.) using an infusion pump at an addition rate of 2.1 mL/h. After 24 h under stirring, BzA (20 µL, 1.6 eq) was added and the solution was subjected to stirring at r.t. for an additional 24 h. The product was precipitated in cold EtOH, filtrated, and dried at 35 °C under a vacuum overnight. Its parameter values were yield = 89%; *M*_w_ (SEC/MALS) = 195.8 kDa; *Ð* (SEC) = 1.21; and *R*_h_ (SEC/VIS) = 6.9 nm.

## 4. Conclusions

We report on a new platform for the synthesis of stable, inert, dispersible, metal-free single-chain nanoparticles (SCNPs) via intramolecular metal-traceless azide–alkyne click chemistry at r.t. The use of the strain-promoted azide−alkyne cycloaddition (SPAAC) technique with *sym*-dibenzo-1,5-cyclooctadiene-3,7-diyne (DIBOD) as an external bifunctional cross-linker molecule, in combination with azide-containing polymeric precursors, provides a new method for synthesizing metal-free SCNPs through intramolecular folding/collapse. We highlight the possibilities of this new synthesis technique by preparing metal-free polystyrene (PS) SCNPs from a copolymer precursor containing 27 mol% of azide functional groups. The successful formation of metal-free PS-SCNPs was confirmed through a powerful combination of techniques, including SEC with triple detection (RI, MALS, and VIS), ^1^H and ^13^C NMR spectroscopy, DLS, FTIR, TGA, and SAXS measurements. In addition to the advantages of these metal-free SCNPs, including their potential use in applications in which the total absence of metal traces is essential, the resulting nanoparticles exhibited significant long-term stability against irreversible aggregation. Finally, it is worth mentioning the potential versatility of this method for the synthesis of long-term-dispersible, metal-free SCNPs from any polymer precursor decorated with azide functional groups.

## Data Availability

The original data are available from the authors upon reasonable request.

## Sample Availability

Samples of the compounds are available from the authors.
